# The Role of Genetic and Non-Genetic Factors in the Occurrence of Cisplatin-Associated Ototoxicity

**DOI:** 10.3390/ijms26104787

**Published:** 2025-05-16

**Authors:** Andreea Iațentiuc, Iustin Mihai Iațentiuc, Otilia Elena Frăsinariu, Sebastian Romică Cozma, Oana Roxana Bitere-Popa, Raluca Olariu, Luminița Mihaela Rădulescu, Ileana Ioniuc, Magdalena Cuciureanu, Mirabela Alecsa, Constantin Guma, Ingrith Crenguța Miron

**Affiliations:** 1 Departament of Mother and Child Medicine, University of Medicine and Pharmacy Gr. T. Popa, 700115 Iași, Romania; andreeacimpan@yahoo.com (A.I.); ileanaioniuc@yahoo.com (I.I.); ingridmiron@gmail.com (I.C.M.); 2 Doctoral School “Grigore T. Popa” University of Medicine and Pharmacy, University Street, No. 16, 700115 Iasi, Romania; iatentiuc_iustin@yahoo.com (I.M.I.); dr.gumaconstantin@gmail.com (C.G.); 3 Surgery II Department, ENT Discipline, “Grigore T. Popa” University of Medicine and Pharmacy, University Street, No. 16, 700115 Iasi, Romania; scozma2005@yahoo.com (S.R.C.); raluca_bcn@yahoo.com (R.O.);; 4 Biomedical Sciences Department, Faculty of Medical Bioengineering, “Grigore T. Popa” University of Medicine and Pharmacy, University Street, No. 16, 700115 Iasi, Romania; oana.bitere@gmail.com; 5 Department of Pharmacology, “Grigore T. Popa” University of Medicine and Pharmacy, 700115 Iasi, Romania; mag.cuciureanu@umfiasi.ro

**Keywords:** genetic factors, non-genetic factors, ototoxicity, cisplatin, adverse effects

## Abstract

There is significant inter-individual variability in the prevalence and severity of cisplatin-induced ototoxicity, which is greatly influenced by genetic and non-genetic factors that predispose the patient to the development of hearing loss. Currently, the focus should be on identifying patients who are more likely to develop ototoxicity based on genetic and non-genetic factors, as therapies to combat ototoxicity are limited or still under study. The severity of hearing loss and the time of its onset may be influenced by certain genetic polymorphisms or the dose administered, age, sex, diet, the administration of other drugs with ototoxic potential, and association with radiotherapy of the head and neck. Knowing the risk factors allows the doctor to manage each case in a personalized manner, preventing hearing damage, especially in the long term. With the help of PubMed and Scopus, we searched for relevant studies documenting the genetic and non-genetic risk in patients treated with cisplatin. This review article is a synthesis of the literature that points out the importance of these factors, encouraging genetic screening and improving quality of life in patients treated with cisplatin.

## 1. Introduction

Cisplatin has been studied over time because, in addition to being successful in curing cancer, it brings with it a few adverse effects that are difficult to prevent. This drug has an expansive toxicity profile that encompasses multiple systems (gastrointestinal, auditory, renal, hematological, etc.) [1] and which considerably influences patients’ quality of life, therapeutic success, and, finally, the patient’s cure rate [2]. The purpose of this review was to identify the main genetic and non-genetic factors that contribute to the ototoxicity of cisplatin.

## 2. Main Side Effects of Cisplatin

**Figure 1:**
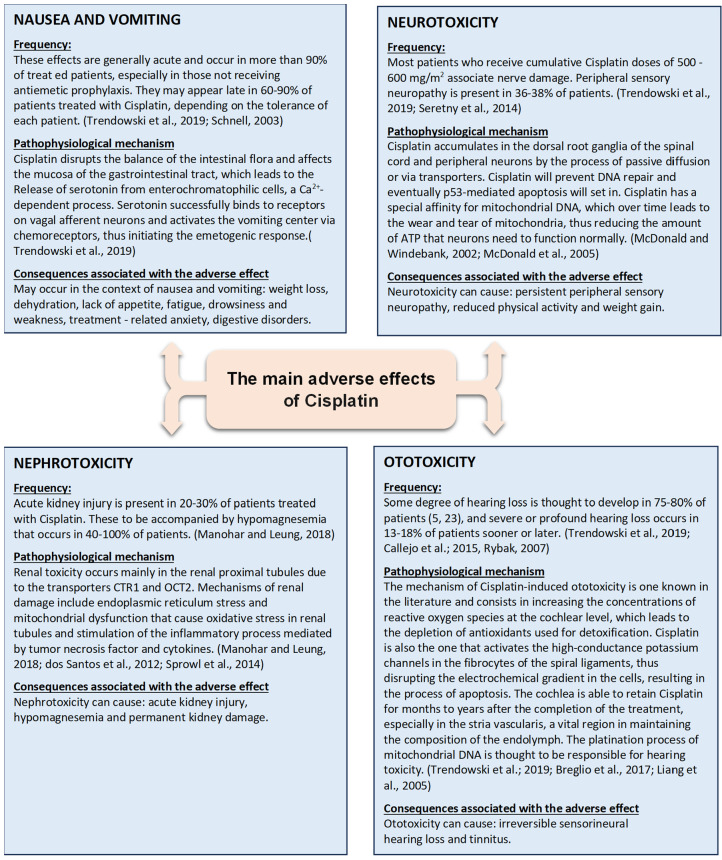
The main side effects of cisplatin [2,3,4,5,6,7,8,9,10,11,12,13].

Figure 1 illustrates the main adverse effects caused by cisplatin and their characteristics.

Among the adverse effects caused by cisplatin, ototoxicity is the most common adverse effect and the most controversial due to its increased incidence (between 13% and 96% depending on the study) and also because of the consequences it brings with it regarding quality of life and social integration [14]. The variation in incidence can be explained by the use of different classifications of ototoxicity (the Brock classification, Muenster classification, Boston classification, Chang classification, ASHA classification, CTCAE classification, and Standard National Cancer Institute classification) [15], the variation of the groups of patients studied and their susceptibilities, or the presence of certain peculiarities, all of which affect the incidence of ototoxicity [14,16].

There is, however, also significant inter-individual variability in the prevalence and severity of cisplatin-induced ototoxicity. In some children, irreversible hearing loss may occur after a single dose of cisplatin, while in other children, hearing loss may not occur even after multiple or increased doses of cisplatin. This can be explained by potential genetic variants that predispose to ototoxicity [17]. The prevalence of hearing loss after cisplatin therapy depends on the cumulative dose and is up to 90% [15].

By ototoxicity, we mean temporary or permanent auditory dysfunction of the inner ear because of treatment with an ototoxic compound (loop diuretics, quinine, aminoglycosides, non-steroidal anti-inflammatory drugs, etc.). The likelihood of inner ear damage will increase when patients receive a combination of two or more ototoxic drugs [1,18].

Cisplatin-induced hearing loss is usually neurosensory, bilateral, irreversible, and progressive, initially affecting high frequencies (4–8 kHz) and then low frequencies, which frequently happens if the dose of cisplatin is increased or combined with another ototoxic drug [14]. The degree of hearing loss differs greatly. Some children have hearing loss at high frequencies, while other children have hearing loss at frequencies lower than 4 kHz, requiring an assistive device to help them. Partial hearing loss that selectively affects certain frequencies deteriorates speech discrimination, with effects on performance in language acquisition and diction in young children and on school performance in older children [19].

Tinnitus affects 15% of the general population, while in the population undergoing treatment with cisplatin, it is present in 40% of cases. Severe forms of tinnitus occur significantly more frequently in patients treated with cisplatin (13–22%) than in the general population (1–2%) [2,20]. Tinnitus can be permanent or transient and can occur with or without hearing loss. In most cases, hearing loss is permanent if it is not treated in time, although sometimes there is partial or sporadic recovery. Cases of unilateral hearing loss are very rare, and they are most often justified by the location of the tumor and the therapeutic interventions performed [1,21].

It is estimated that more than half a million people suffer from moderate or severe hearing loss each year due to treatment with various ototoxic agents. The situation is extremely complicated because, on the one hand, they try to support the therapeutic efficacy of the antineoplastic treatment, while on the other hand, they try to prevent side effects. Ototoxicity caused by platinum compounds, but especially by cisplatin, can persist even throughout the patient’s life [22]. This is why it is important to understand the risk factors that influence the occurrence of ototoxicity to intervene in a timely manner and thus prevent hearing loss, especially in childhood.

## 3. Genetic and Non-Genetic Factors in Cisplatin Ototoxicity

To understand the mechanisms of cisplatin ototoxicity, it is very important to consider the impact that genetic or non-genetic factors have on this process. Given that there is no clarity on the “miracle drugs” that could mitigate ototoxicity but simply leads and studies that try to implement such therapies, the only handy and safe way remains to identify individuals who are more likely to develop adverse effects to cisplatin treatment based on genetic and non-genetic factors [2].

Hearing loss is caused by several factors and can occur in all age groups. The severity of hearing loss and the time of its occurrence vary from one case to another, being mainly dependent on the dose administered (the higher the cumulative dose, the more pronounced the ototoxic effect), the duration of treatment, the number of cycles administered, and the way they are administered. Other factors that increase the risk of ototoxicity are exposure to loud noises during treatment, combination with other ototoxic chemicals or drugs, pre-existing renal pathologies that could influence treatment with cisplatin, or pre-existing hearing loss that may worsen if not detected before starting treatment with cisplatin. Among the physiological factors influencing the ototoxicity of cisplatin is the age of the patient (young children and adults over 46 years of age have more significant hearing impairments). In addition, genetic factors weigh heavily in the ototoxic process of cisplatin, along with environmental factors [1].

### 3.1. Genetic Factors

Genetic risk factors for ototoxicity caused by cisplatin, especially in children, have become a hotly debated topic, with numerous studies trying to prove that there are genes that could influence the onset of ototoxicity, with some children being more likely to acquire it. The single-nucleotide polymorphisms (SNPs) of genes that have been studied over time are especially those that encode enzymes or proteins (glutathione S-transferase = GTS; excision repair cross-complementing group = ERCC; thiopurine S-methyltransferase = TPMT; and catechol-O-methyltransferase = COMT). The side effects of a drug are influenced by the intracellular absorption and metabolization of the drug, as well as the detoxification and elimination process. Certain genetic polymorphisms that are involved in encoding enzymes or proteins act during the above stages of the drug and may increase the risk of toxicity [14].

A study in 72 children treated with cisplatin looked at genetic factors that could contribute to ototoxicity. The study evaluated six single-nucleotide polymorphisms (SNPs) as potential genetic factors using PCR (polymerase chain reaction). These polymorphisms are *ERCC1 rs11615*, *GSTP1 rs1138272*, *GSTP1 rs1695*, *LRP2 rs2075252*, *TPMT rs12201199*, and *COMT rs9332377* [14,15].

*LRP2* (the low-density lipoprotein receptor gene) is involved in encoding a protein receptor that has been identified in renal proximal tubular cells and the marginal cells of the vascular stria in the cochlea and is thought to be responsible for the intracellular uptake of cisplatin and aminoglycosides. Some studies have established a relationship between *LRP2* and the onset of ototoxicity [15,23], while others have failed to do so [24]. The Riedman study observed the link between the A allele of *LRP2 rs2075252* and the occurrence of cisplatin-induced ototoxicity in research conducted on 50 children [23]. The fact that some studies found a link between *LRP2* and ototoxicity while other studies did not is due to certain risk factors that have or have not been taken into account, such as different ethnic groups, younger or older patients, the use of other drugs with ototoxic potential, cranial irradiation, and factors that could influence the results [14]. The *LRP2* gene, or megalin, is the only gene associated with Donnai–Barrow syndrome. This syndrome is characterized by craniofacial abnormalities, ocular abnormalities, sensorineural deafness, and developmental delay. There are also associations of the *LRP2* gene with diabetic nephropathy, Lowe syndrome, Dent’s disease, Alzheimer’s disease, and gallstones [15,25].

Glutathione-S-transferases (GSTs) are antioxidant enzymes that protect the cell by scavenging oxygen free radicals. Single-nucleotide polymorphisms (SNPs) in different isoforms of GST (*GSTM*, *GSTP*, and *GSTT*) can contribute to a decrease in the enzymatic activity of cells, leading to the occurrence of ototoxicity [26]. In the study conducted by Olgun and collaborators, it was observed that the mutant genotype of *GSTP1 rs1695* is related to the ototoxicity of cisplatin [14]. Lui and collaborators also identified an ototoxic effect [27]. However, two studies did not find a link between this genotype and ototoxicity [15,28]. The *GSTP1 rs1695* genotype appears to be a genetic risk factor, but larger patient studies are needed to confirm this hypothesis [14]. Peter et al. observed that a single-nucleotide polymorphism in the *GSTM3 B* allele is associated with a lower risk of ototoxicity in the population with this polymorphism compared to the normal population [29]. *GSTM1 del* shows an insignificant association with ototoxicity, which has been demonstrated by several studies [15,24,27,28,30]. For *GSTT1 del*, the situation is a bit ambiguous, since there are studies that associate it with a partial ototoxic effect [27,31], a partial otoprotective effect [24], and studies that do not find an exact association between it and the ototoxicity induced by cisplatin [15,28,30].

*TPMT* and *COMT* encode several enzymes involved in the inactivation of azathioprine metabolites. When these enzymes are inactivated, the amount of cisplatin that reaches the cross-links of DNA will be increased. Ross et al. found that there is a link between the SNPs of the *TPMT* and *COMT* genes and cisplatin-driven ototoxicity [32]. TPMT is a methyltransferase whose low activity can lead to myelosuppression, gastrointestinal intolerance, pancreatitis, and hypersensitivity. *TPMT rs12201199* has been associated with reduced TPMT enzyme activity and higher toxicity driven by antineoplastic drugs [33,34]. Several polymorphisms of *TPMT* are shown in Table 1, depending on the effects discovered and the studies that mention them. Studies indicate that mutations in the *COMT* genes are linked to sensorineural hearing loss due to the degeneration of hair cells. Additionally, individuals with the *COMT Met allele* have a reduced risk of hearing loss, likely due to the protective effect of dopamine on the auditory system. However, there is evidence supporting the beneficial role of catecholamines in the auditory system [15,35].

*ACYP2* (the acylphosphatase 2 gene) encodes the enzyme involved in catalyzing phosphate hydrolysis in membrane pumps, especially ATP-ase Ca^2+/^Mg^2+^ from the sarcoplasmic reticulum of skeletal muscle. In the cochlea, *ACYP2* is involved in ATP-dependent Ca^2+^ signaling, an extremely important process in the development of hair cells [2]. Three studies confirmed the association of *ACYP2* with hearing loss, one in 156 patients with osteosarcoma [36], another in 149 patients with pediatric cancer [17], and the last in testicular cancer patients [37]. *ACYP2* is involved in the encoding of acylphosphatase-2 at the cochlear level, thus affecting the homeostasis of Ca^2+^ ions at the membrane level. Thiesen et al. also noted that there is an association between *ACYP2* and cisplatin-induced ototoxicity, although further studies are needed to understand the biological link underlying this association [17]. Tserga et al. argue that based on the study conducted, there is a link between *rs1872328* on *ACYP2* (which is involved in calcium homeostasis) and an increased risk of ototoxicity [15].

*SOD2 rs4880* is a polymorphism associated with an increased risk of ototoxicity [38]. SOD2 (superoxide dismutase 2) catalyzes the metabolism of the superoxide anion (highly toxic) to hydrogen peroxide (less toxic). SNP *rs4880* results in the replacement of alanine with valine, and this polymorphism increases the catalytic activity of SOD2, resulting in the accumulation of excess hydrogen peroxide and the generation of ROS (reactive oxygen species). In this way, altered mitochondrial function at the cochlear level will contribute to increasing the ototoxicity threshold of cisplatin [19].

There are also a few genes that have been shown to have an otoprotective effect against the toxicity caused by cisplatin. Khokhrin et al. concluded that the single-nucleotide polymorphism of epoxide hydrolase 1 (*EPXH1 rs2234922*) would have an otoprotective effect [39]. Spracklen et al. also identified two single-nucleotide polymorphism predictors of otoprotection in cisplatin treatment: *rs6721961* of the NFE2L2 gene, with a role in the protection of cells against oxidative stress, and *rs10950831* of the ABCB5 (ATP-binding cassette subfamily B member 5) gene, which contributes to the efflux of cellular cisplatin [40].

*SLC22A2* (solute carrier family 22 member 2) is involved in the encoding of CTR1 (copper transporter protein 1), with a role in the absorption of cisplatin in cochlear hair cells. Cisplatin accumulates at the level of the vascular stria in the cochlea, and this genetic polymorphism contributes through its role on the transport protein to an improvement in the function of the cochlear vascular stria affected by cisplatin [15].

*ABCC3 rs1051640* (ATP-binding cassette subfamily C member 3) is involved in acquiring resistance to several drugs. A genetic polymorphism is involved in regulating the efflux of organic and xenobiotic anions. Its expression in cancer cells correlates with resistance to cisplatin. The mechanism of action at the cochlear level is not yet very well-outlined, with some studies suggesting that it acts upstream of GST [41].

Tserga E. et al. [15] conducted a systematic review in which they analyzed several studies on genes that could contribute to the development of cisplatin-induced ototoxicity. From the analysis of various studies, they concluded that there are genes with ototoxic potential, genes with otoprotective potential, and neutral genes that are not associated with any effects on the auditory system. Tserga E. et al. [15] concluded that there are genes with an insignificant association with ototoxicity, including *CTR1 rs10981694*, *GSTM1 del*, *GSTT1 del*, *GSTP1 rs1695*, *SLC16A5 rs4788863*, *XPC rs2228001* (Xeroderma pigmentosum complementation group C), and *XPD rs1799793* (Xeroderma pigmentosum complementation group D); genes with a significant association with ototoxicity, such as *LRP2 rs2075252*, *LRP2 rs4668123*, *TPMT rs12201199*, *TPMT rs1142345*, *TPMT rs1800460*, *COMT rs9332377*, *ACYP2 rs1872328*, and *SOD 2 rs4880*; and genes with otoprotective potential, including *GSTM3 RS1799735*, *SLC22A2 RS316019*, and *ABCC3 RS1051640*. Genes with ototoxic potential are involved in the regulation of antioxidants, neurotransmission, or are related to auditory function [15]. Table 1 aims to highlight the genes studied and the effect they may have on the auditory system, based on the aforementioned study (Tserga et al., 2019) [15] and the literature review. The effects of the genes will be specified according to the results of each analyzed study.

**Table 1:** Genes involved in the processes of ototoxicity and otoprotection.

The Gene Involved	The Effect	The Study and the Authors Who Mention It	Remarks
**The copper transporter protein 1 *(CTR1) rs10981694***	Insignificant association with ototoxicity	(Tserga et al., 2019) [15] (Lanvers-Kaminsky et al., 2015) [42] (Xu et al., 2012) [43]	One way in which cisplatin enters the cochlea is through the copper transporter 1 (CTR1). In the cochlea, CTR1 is in the outer hair cells, inner hair cells, the stria vascularis, and the neurons of the spiral ganglia, facilitating the drug’s entry at these sites and ultimately leading to cellular apoptosis. [42] This process could interfere with the synthesis of functional proteins and reduce the efficiency of gene translation. Any genotypic polymorphism may cause a structural modification of the CTR1 protein, thereby affecting its transporter function. Meanwhile, genetic mutations may also contribute to the regulation of CTR1 expression. The analyzed studies do not find a significant association with the risk of ototoxicity in patients treated with cisplatin. However, the study by Xu et al. [43] concludes that there may be an association with cisplatin-induced ototoxicity in Asian and Caucasian patients. Molecular and cellular research is needed to explore how CTR1 mutations affect the absorption, accumulation, efficacy, and response to Cisplatin treatment, as there are few studies available, and their conclusions are contradictory.
***GSTM1 del***	Insignificant association with ototoxicity	(Tserga et al., 2019) [15] (Lui et al., 2018) [27] (Choeyprasert et al., 2013) [24] (Barahmani et al., 2009) [30] (Oldenburg et al., 2007) [28]	One of the mechanisms by which Cisplatin causes ototoxicity is the generation of reactive oxygen species, which induce oxidative stress in the cochlea. This process is counteracted by antioxidant enzymes, specifically glutathione S-transferases (GSTs), which prevent this effect. For GSTM1, all the studies we analyzed found an insignificant association with ototoxicity. The absence of both GSTM1 and GSTT1 may further increase the risk of ototoxicity. Some patients with a combined null GSTM1 + null GSTT1 genotype developed severe hearing loss compared to those who had at least one functional gene.
***GSTT1 del***	Insignificant association with ototoxicity	(Tserga et al., 2019) [15] (Barahmani et al., 2009) [30] (Oldenburg et al., 2007) [28]	GSTT1 helps neutralize toxic compounds by conjugating them with glutathione. In the absence of GSTT1 (null GSTT1 genotype), cisplatin elimination is less efficient, which can lead to increased drug accumulation in the hair cells of the inner ear, thereby promoting cell death and hearing loss. Studies have shown that patients with a *null GSTT1* genotype are more prone to severe ototoxicity following cisplatin treatment. In a murine model (CBA/CaJ mice), it was demonstrated that the simultaneous absence of GSTT1 and GSTM1 exacerbates cisplatin-induced hearing loss, suggesting a protective role of these genes against the drug’s toxic effects. Since the process of evolution had no opportunity to adapt to the toxic effects of chemical compounds introduced during industrialization and modern medications, genetically deleted GST isoenzymes may play a role in either the toxification or detoxification of synthetic chemicals or drugs. This explains the contradictory results of studies, in addition to differences in interpretation scales and the influence of various non-genetic factors (such as sex, age, cancer type, treatment duration, etc.) that can impact outcomes.
	Partial ototoxic effect	(Lui et al., 2018) [27] (Talach et al., 2016) [31]	
	Potential otoprotective effect	(Choeyprasert et al., 2013) [24]	
***GSTP1 rs1695***	Insignificant association with ototoxicity	(Tserga et al., 2019) [15] (Oldenburg et al., 2007) [28]	GSTP1 (glutathione S-transferase P1) is an enzyme that, like GSTM1 and GSTT1, helps detoxify toxic compounds, including cisplatin, by conjugating them with glutathione. Polymorphic variants of GSTP1, particularly *rs1695* (which results in the substitution of the amino acid isoleucine with valine at position 105), can influence enzymatic activity and the efficiency of detoxification processes, which may affect susceptibility to cisplatin-induced ototoxicity [14, 27]. Oldenburg et al. [28] suggest that alterations in intracellular apoptosis pathways could be an additional explanation for the varying detoxification efficiencies: GSTP1 monomers bind to and thus inactivate stress-inducible Jun N-terminal kinase (JNK). Oxidative stress releases GSTP1 from JNK, which in turn activates the expression of GSTP1 and other genes involved in apoptosis and cytoprotection. Inhibition of JNK in guinea pigs treated with cisplatin increased ototoxicity. Hypothetically, protection against cisplatin-induced toxicities could be due to the less efficient inactivation of JNK by the GSTP1 enzyme [28]. The other studies analyzed reveal a clear association with cisplatin-induced ototoxicity.
	Ototoxic effect	(Lui et al., 2018) [27] (Olgun et al., 2016) [14]	
	
***SLC16A5 rs4788863***	Insignificant association with ototoxicity	(Tserga et al., 2019) [15] (Lui et al., 2018) [27]	*SLC16A5 rs4788863* is a genetic polymorphism associated with the *SLC16A5 gene*, which is part of the monocarboxylate transporter family. Genes in this family are involved in the transport of important substances such as lactate, pyruvate, and other metabolites involved in cellular metabolism. Specifically, SLC16A5 is a transporter involved in the exchange of ions and metabolites, playing a crucial role in cellular metabolism, including in auditory cells [15]. Regarding hearing, SLC16A5 may be involved in regulating cellular metabolism in auditory cells of the inner ear, which are sensitive to oxidative stress and toxins, including cisplatin. Since SLC16A5 is involved in the transport of substances, a genetic variant that alters the activity of this transporter could influence the response of auditory cells to ototoxic drugs such as cisplatin. Studies exploring the link between *SLC16A5 rs4788863* and ototoxicity are still ongoing, but it is hypothesized that genetic variants at this level may affect the susceptibility of auditory cells to cisplatin-induced damage. In general, polymorphisms that affect transport and cellular metabolism are often linked to cellular responses to oxidative stress and environmental toxins, with cisplatin being one such agent. It is important to note that studies on the link between *SLC16A5 rs4788863* and ototoxicity are limited, and associations may vary depending on various factors.
	Otoprotective effect	(Drögemöller et al., 2017) [44]	
***XPC rs2228001***	Insignificant association with ototoxicity	(Tserga et al., 2019) [15] (Lui et al., 2018) [27] (Lopes-Aguiar et al., 2016) [45] (Caronia et al., 2009) [46]	The *XPC genes* are involved in DNA repair through the nucleotide excision repair (NER) mechanism, a process that helps repair damage caused by toxic chemicals such as cisplatin. *XPC rs2228001* is a polymorphism in a non-coding region of the *XPC gene* [45]. XPC is crucial for detecting and repairing cisplatin-induced DNA damage. The *rs2228001* variant may affect the efficiency of DNA repair, and individuals with certain variants of this polymorphism may have a reduced ability to repair DNA, thereby increasing the risk of toxic effects, including ototoxicity. Studies have suggested that polymorphisms in the *XPC gene*, including *rs2228001*, could be associated with an increased risk of ototoxicity following cisplatin treatment, as a less efficient repair system may allow DNA damage to accumulate in auditory cells, leading to their death and hearing loss [27]. However, the association is complex and may vary depending on other genetic or environmental factors. For example, some patients with the *rs2228001* variant may not experience severe ototoxicity, while others with a different genetic profile may suffer more severe effects.
***XPD rs1799793***	Insignificant association with ototoxicity	(Tserga et al., 2019) [15] (Lopes-Aguiar et al., 2016) [45]	The *XPD genes* (also known as ERCC2) are also essential for DNA repair through the excision of lesions, being involved in the same nucleotide excision repair (NER) mechanism. *XPD rs1799793* is a polymorphism that affects the function of the *XPD gene*, resulting in a change in the efficiency of the DNA repair process. XPD is involved in correcting DNA damage caused by toxic agents, including cisplatin. The *rs1799793* variant (a polymorphism affecting the activity of the XPD enzyme) can modify the efficiency of this repair process. Individuals with certain variants of *rs1799793* may have a slower or less efficient DNA repair system, which can lead to a greater accumulation of DNA damage in auditory cells, particularly after cisplatin treatment [45]. Studies have suggested that the *rs1799793* variant may be associated with an increased risk of ototoxicity, as a reduced DNA repair capacity may promote the accumulation of damage in the inner ear. Additionally, the effects may vary depending on the specific type of polymorphism and other genetic or environmental factors. Some research has shown significant associations between the *XPD rs1799793* polymorphism and hearing loss following cisplatin treatment. The study conducted by Lui et al. (2018) [27] found a significant association with ototoxicity in a study involving 106 children treated with platinum derivatives, using the Brock criteria.
	Ototoxic effect	(Lui et al., 2018) [27]	
***LRP2 rs2075252***	Insignificant association with ototoxicity	(Choeyprasert et al., 2013) [24]	LRP2, also known as megalin, is a transmembrane receptor that plays an important role in the process of endocytosis and the transport of essential substances such as lipids, vitamins, hormones, and metabolites. Regarding *LRP2 rs2075252*, it is a polymorphism located in a non-coding region of the *LRP2 gene*. Typically, polymorphisms found in non-coding regions of genes can influence gene expression or regulate its function, even if they do not directly alter the structure of the encoded protein. Available studies and research suggest that the *LRP2 rs2075252* variant may play a significant role in the process of ototoxicity by affecting the efficient elimination of toxic substances from the body [15,23]. Choeyprasert et al. [24] examined several genetic variants and their impact on sensitivity to drug-induced ototoxicity, such as cisplatin. Choeyprasert et al. [24] did not find a strong association between *rs2075252* and ototoxicity, but they suggested that variants in LRP2, including *rs2075252*, might have an indirect role by influencing the capacity to eliminate toxic substances, thus affecting the risk of auditory damage. In this context, *rs2075252* does not appear to be directly associated with an increased risk of ototoxicity, but there may be possible indirect implications, particularly concerning detoxification and the elimination of toxic substances from the body.
	Ototoxic effect	(Tserga et al., 2019) [15] (Riedemann et al., 2008) [23]	
***LRP2 rs4668123***	Insignificant association with ototoxicity	(Choeyprasert et al., 2013) [24]	*LRP2 rs4668123* is another polymorphism associated with the *LRP2 gene*, which may affect the functioning of this protein, indirectly influencing the body’s response to toxins, including cisplatin. Regarding *LRP2 rs4668123*, research shows a clearer association with ototoxic effects, particularly in how this polymorphism influences the transport and metabolism of toxic substances. This suggests that *rs4668123* may have a more significant impact on the body’s response to toxins. Riedemann et al. [23] investigated polymorphisms in genes involved in detoxification processes, including *LRP2*, and found a stronger correlation between variants in this gene and cisplatin-induced ototoxicity. Their study indicated that *rs4668123* might alter the efficiency of toxic substance transport and reduce the body’s ability to eliminate cisplatin, leading to increased accumulation in auditory cells and, consequently, a higher risk of hearing loss. According to this study, *rs4668123* was associated with a greater risk of ototoxicity due to its effects on the elimination and transport of toxic substances, including cisplatin. Based on the studies by Choeyprasert et al. (2013) [24] and Riedemann et al. (2008) [23], LRP2 rs4668123 appears to be more ototoxic than *LRP2 rs2075252*, showing a stronger association with the risk of cisplatin-induced ototoxicity.
	Ototoxic effect	(Tserga et al., 2019) [15] (Riedemann et al., 2008) [23]	
***TPMT rs12201199***	Insignificant association with ototoxicity	(Thiesen et al., 2017) [17] (Hagleitner et al., 2014) [47] (Yang et al., 2013) [48]	Polymorphisms in the ***TPMT*** (*thiopurine S-methyltransferase*) gene have been studied for their possible association with cisplatin-induced ototoxicity, but the results remain mixed. **TPMT** is an enzyme involved in the metabolism of thiopurines, but it also plays a role in drug detoxification, including compounds derived from cisplatin. The effect of ***TPMT*** polymorphisms on ototoxicity is complex and context-dependent. Some studies do not find a clear association with the ototoxicity process, while others suggest an increased risk. These differences may be due to factors such as the studied population, cisplatin dosage, genetic interactions, and the analytical methods used. A broader approach, including meta-analytical studies and functional analyses, is needed to clarify the exact way in which ***TPMT*** influences ototoxicity. Currently, ***TPMT*** cannot be considered a definitive genetic marker for ototoxicity, but it remains a candidate for further research due to its potential role in predicting the risk of hearing loss in patients treated with cisplatin. ***TPMT rs12201199*** appears to have the strongest association with ototoxicity according to studies. This polymorphism could serve as a useful genetic marker for identifying patients at higher risk. ***TPMT rs1142345*** may influence ototoxicity, but the evidence is mixed. It is possible that this variant plays a more significant role when combined with other genetic factors. ***TPMT rs1800460*** does not have a clear association with ototoxicity, and some studies even suggest a potential protective effect (Ross et al., 2009) [32]. Overall, TPMT variants likely influence the metabolism of cisplatin, but further studies are needed to confirm the exact impact of these polymorphisms on ototoxicity.
	Ototoxic effect	(Tserga et al., 2019) [15] (Pussegoda et al., 2013) [49] (Ross et al., 2009) [32]	
***TPMT rs1142345***	Insignificant association with ototoxicity	(Thiesen et al., 2017) [17] (Hagleitner et al., 2014) [47] (Yang et al., 2013) [48]	
	Ototoxic effect	(Tserga et al., 2019) [15] (Pussegoda et al., 2013) [49] (Ross et al., 2009) [32]	
***TPMT rs1800460***	Insignificant association with ototoxicity	(Thiesen et al., 2017) [17] (Hagleitner et al., 2014) [47] (Yang et al., 2013) [48] (Ross et al., 2009) [32]	
	Ototoxic effect	(Tserga et al., 2019) [15] (Pussegoda et al., 2013) [49]	
***COMT rs9332377***	Insignificant association with ototoxicity	(Thiesen et al., 2017) [17] (Pussegoda et al., 2013) [49] (Yang et al., 2013) [48]	**COMT** (catechol-O-methyltransferase) is an enzyme involved in the metabolism of catecholaminergic neurotransmitters (dopamine, epinephrine, and norepinephrine) and cellular detoxification processes. **COMT** plays a role in regulating oxidative stress, and genetic variants of this gene may influence the sensitivity of auditory cells to damage induced by cisplatin. The ***rs9332377*** polymorphism is in a regulatory region of the ***COMT*** gene and may affect the expression levels of the enzyme. The ***COMT rs9332377*** polymorphism has been investigated in several studies on cisplatin-induced ototoxicity, but the results are mixed. Some studies suggest a possible association, while others do not confirm a significant effect. Hagleitner et al. [47] suggest that ***rs9332377*** may have a protective effect against cisplatin-induced ototoxicity by reducing oxidative stress and protecting auditory cells. However, this finding has not been consistently confirmed by other studies, indicating that the protective effect may be influenced by additional factors such as population differences, genetic interactions, and the dosage of cisplatin. Overall, ***COMT rs9332377*** remains an interesting polymorphism, but further studies are needed to confirm its role.
	Ototoxic effect	(Tserga et al., 2019) [15] (Talach et al., 2016) [31] (Ross et al., 2009) [32]	
	Potential otoprotective effect	(Hagleitner et al., 2014) [47]	
***ACYP2 rs1872328***	Ototoxic effect	(Tserga et al., 2019) [15] (Drögemöller et al., 2018) [37] (Thiesen et al., 2017) [17] (Vos et al., 2016) [36] (Xu et al., 2015) [50]	The *ACYP2 rs1872328* polymorphism has been investigated in multiple studies for its association with cisplatin-induced ototoxicity, and evidence suggests that it plays a significant role in genetic susceptibility to hearing loss. ***ACYP2*** is a gene involved in energy metabolism and cellular signalling. Studies suggest that the *rs1872328* polymorphism affects biochemical pathways related to the survival of hair cells in the inner ear, making them more vulnerable to damage caused by cisplatin. The study by Xu et al. was one of the first to identify ***ACYP2 rs1872328*** as a genetic factor associated with cisplatin-induced ototoxicity. ***ACYP2*** may influence metabolic pathways that affect the sensitivity of hair cells to oxidative stress, leading to cell death and ototoxicity. This study paved the way for further research, indicating that patients carrying the *rs1872328* variant have a higher risk of ototoxicity. The study by Vos et al. [36] attempted to replicate Xu et al.’s findings [50] in a different cohort. Although they confirmed a certain association between ***rs1872328*** and ototoxicity, the effect was weaker than in the previous study. The differences may be due to ethnic variations and the doses of cisplatin administered to patients. The study by Thiesen et al. [17] investigated multiple polymorphisms in the context of cisplatin-induced ototoxicity. They did not find a clear association between ***rs1872328*** and ototoxicity but noted that the trend observed in other studies warrants further investigation. The study did not confirm a strong association but also did not rule out the possibility that ***rs1872328*** plays a role. The study by Drögemöller et al. [37] analyzed genetic factors involved in ototoxicity in a large and diverse population. They identified ***rs1872328*** as a significant marker of ototoxicity. The study by Tserga et al. [15] was a meta-analysis evaluating multiple genes associated with ototoxicity. It confirmed that ***ACYP2 rs1872328*** is associated with an increased risk of ototoxicity, supporting the initial conclusions of Xu et al. (2015) [50]. The polymorphism may affect the cell cycle and metabolic pathways essential for the protection of auditory cells. Tserga et al. [15] suggest that ***rs1872328*** could be used as a genetic marker to identify patients at higher risk of hearing loss before undergoing cisplatin treatment. Overall, ***rs1872328*** appears to be one of the most well-studied biomarkers of cisplatin-induced ototoxicity, but its effect may depend on factors such as the cisplatin dose, genetic background, and interactions with other genetic and environmental factors.
***SOD 2*** **(superoxide dismutase 2 mitochondrial) *rs4880***	Insignificant association with ototoxicity	(Lui et al., 2018) [27]	The ***rs4880* polymorphism** of the ***SOD2*** gene has been investigated in the context of cisplatin-induced ototoxicity, particularly in pediatric patients with medulloblastoma. A significant study in this area is that of **Brown et al.**, [38] which analyzed the association between this polymorphism and hearing loss in children treated with cisplatin. In this study, it was found that the **C allele** of the ***rs4880 polymorphism*** is associated with an increased risk of ototoxicity. Specifically, patients who inherited this allele were more likely to experience significant hearing loss following cisplatin treatment. The proposed mechanism suggests that the **Ala variant** (encoded by the C allele) increases the activity of the **MnSOD enzyme**, which may result in an excessive accumulation of hydrogen peroxide in cochlear cells, thereby contributing to oxidative stress and hearing damage. It is important to note that while this study provides evidence of an association between *rs4880* and ototoxicity, further research is needed to confirm these findings and to fully understand the underlying mechanisms involved.
	Ototoxic effect	(Tserga et al., 2019) [15] (Brown et al., 2015) [38]	
***GSTM3 rs1799735***	Insignificant association with ototoxicity	(Lui et al., 2018) [27]	In a study by Peters et al. [29], an association with otoprotection was observed for the *GSTM3 polymorphism*. It is indeed believed that variations in *GSTM3* alter susceptibility to potential carcinogens and toxins. There is research suggesting that *GSTM3 rs1799735* may be an otoprotective factor under certain conditions, but it has not been fully clarified by a single study.
	Otoprotective effect	(Tserga et al., 2019) [15] (Peters et al., 2000) [29]	
***SLC22A2 rs316019* (drug efflux transporter)**	Insignificant association with ototoxicity	(Tserga et al., 2019) [15] (Spracklen et al., 2017) [40]	The single-nucleotide polymorphism *rs316019* in the *SLC22A2 gene*, also known as Ala270Ser, affects the function of the organic cation transporter 2 (hOCT2). This transporter is essential for the elimination of certain drugs and metabolites through the kidneys. Both the study by Lanvers-Kaminsky et al. [42] and that by Spracklen et al. [40] highlight the importance of the *rs316019 polymorphism* in modulating hOCT2 function. These studies provide valuable insights into how the *rs316019* variant can influence drug response and the risk of side effects, paving the way for personalized therapeutic strategies based on the patient’s genetic profile. In theory, if *rs316019* modulates the transport of cisplatin and thus reduces its accumulation in auditory tissues, this polymorphism could protect against ototoxicity. This would imply an otoprotective effect. However, direct evidence for such an effect is still lacking.
	Otoprotective effect	(Lanvers-Kaminsky et al., 2015) [42]	
***ABCC3 rs1051640***	Insignificant association with ototoxicity	(Spracklen et al., 2017) [40] (Pussegoda et al., 2013) [49]	The *ABCC3 rs1051640* polymorphism is part of the *ABCC3 gene*, which encodes an ATP-binding cassette (ABC) family membrane transporter called MRP3 (multidrug resistance protein 3). MRP3 is involved in the transport of various toxic substances, including drugs, across cell membranes, particularly in the liver, kidneys, and other tissues. Regarding ototoxicity and otoprotection, studies directly addressing *ABCC3* and the *rs1051640 polymorphism* are limited. Based on its role in expelling toxic substances and various items of evidence suggesting that MRP3 transporters can reduce toxin accumulation, *ABCC3 rs1051640* could be associated with an otoprotective effect if this variant leads to increased MRP3 transporter activity, promoting faster elimination of cisplatin from cochlear cells and thus reducing the risk of damage [40,49].
	Otoprotective effect	(Tserga et al., 2019) [15]	

By identifying these genes in children with potential ototoxic treatment indications, the magnitude of side effects could be limited by choosing a less ototoxic alternative therapy.

However, there are still many children treated with cisplatin who develop hearing loss and yet are not pre-emptively identified with the help of genetic testing. Investigations and studies that contribute to improving these areas provide valuable information about the metabolism and sensitivity of cisplatin, which, over time, allow a more optimal dosage of the drug and, of course, a decrease in the risk of hearing loss in children with cancer.

### 3.2. Non-Genetic Factors

**Figure 2:**
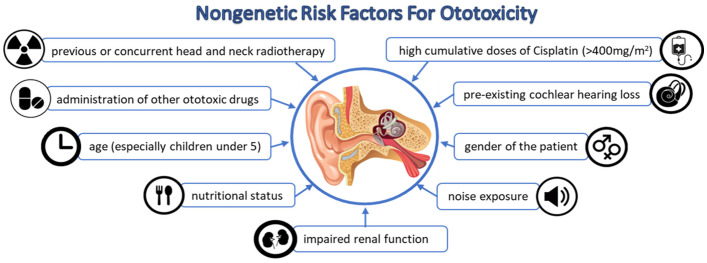
Non-genetic risk factors.

Among the non-genetic risk factors that appear to influence the occurrence of cisplatin-induced ototoxicity, the following have been mentioned: radiotherapy to the anterior or concomitant head and neck region, high cumulative doses of cisplatin (>400 mg/m^2^), the age (especially children younger than 5 years) and sex of the patient, administration of other ototoxic drugs (aminoglycosides, vancomycin, amphotericin B, carboplatin, vincristine, and furosemide), poor renal function, nutritional status, and pre-existing cochlear hearing loss [14,15,17,19]. (Figure 2) illustrates the main non-genetic factors underlying the ototoxicity process.

#### 3.2.1. Age and Sex of the Patient

Age plays an important role in the occurrence of cisplatin-induced ototoxicity due to the cochlear peculiarities induced by this factor. Age influences the risk of occurrence, the mechanisms of production, and even the severity of ototoxicity. There are numerous studies attesting to the increased susceptibility to ototoxicity in childhood [19,51,52,53]. In children (especially young children), the cochlea is in the process of development, which makes it more sensitive to the action of ototoxic drugs. The outer hair cells have not reached maturity, which makes them more susceptible to oxidative damage and the accumulation of ROS (reactive oxygen species) in the inner ear. The metabolism of children differs greatly from that of adults, affecting the distribution, metabolism, and excretion of cisplatin. A faster or immature metabolism can lead to high concentrations of cisplatin at the cochlear level. The natural mechanisms of the repair of cellular DNA affected by ROS, or those of detoxification, are less effective in children, thus increasing the likelihood of cell damage. Unlike adults, children are more prone to progressive hearing loss after treatment with cisplatin has ended [11,53].

Adults have more efficient mechanisms for repairing cellular DNA, but also complex and mature mechanisms for managing oxidative stress. The ability to metabolize and excrete cisplatin in adults is more stable, which limits its accumulation in the cochlea. However, the cumulative total dose of cisplatin remains a critical factor in the development of ototoxicity, and adults who receive high doses or have additional risk factors (such as renal failure or exposure to other ototoxic drugs) may develop similar ototoxicity to children or the elderly [52,53].

The elderly are also susceptible to ototoxicity for several physiological and genetic reasons. Presbycusis (hearing loss with age) may be exacerbated by the ototoxicity of cisplatin, leading to accelerated hearing loss and early complications. With age, changes in the cochlea occur (decreases in the number of hair cells, alterations in their functions, and the degeneration of auditory neurons), which make the inner ear more sensitive to the action of external factors. In the elderly, there is a natural decrease in antioxidant enzymes, and the cumulative effects of oxidative stress and chronic aging-related inflammation make cochlea cells more susceptible to oxidative damage. Chronic diseases such as hypertension, diabetes, and kidney failure exacerbate the toxicity of cisplatin on the cochlea, and the use of other drugs, including ototoxic ones, is more common in the elderly, which may increase the risk of cisplatin-induced ototoxicity [54,55].

Gender plays an important role in susceptibility to cisplatin-induced ototoxicity, and biological differences between men and women can influence how toxicity manifests itself and evolves in the inner ear. These differences are attributed to a variety of factors, such as hormones, genetic factors, differences in drug metabolism, and immune response. Male gender and aminoglycoside co-treatment appear to have a significant impact on the occurrence of ototoxicity in children and adolescents treated with cisplatin. Most studies support the fact that male gender is a risk factor for ototoxicity caused by cisplatin [14,19,56]. One of the reasons why females have a lower risk of developing ototoxicity is the possible otoprotective effect of estrogen. It has been found in several studies that estrogen has a favorable effect on hearing, and estrogen receptors have been discovered in the cells of the vascular stria [14,57,58]. However, after menopause, when estrogen levels drop, the risk of ototoxicity may increase. Boys have a four-times-higher risk of hearing loss following treatment with cisplatin [19].

Testosterone has a lesser protective effect on cochlear cells compared to estrogen, which may explain men’s increased predisposition to ototoxicity. Some studies support that testosterone may increase the risk of inflammation and oxidative stress, which could accentuate cisplatin-induced damage to the cochlea [59,60]. Genetic factors and immunity may also play an important role in how men and women respond to cisplatin. In men, the expression of certain genes that regulate inflammation and apoptosis may be stronger, which may accelerate cochlear damage upon exposure to cisplatin. Women’s immune systems tend to be more active than men’s, which could boost protection against some inflammatory effects of cisplatin but could also increase the risk of autoimmune reactions that worsen ototoxicity. Men may exhibit a different inflammatory response, which could contribute to the accelerated apoptosis of sensitive cells in the cochlea under the effect of cisplatin-induced stress [19,59,60].

The study led by Yancey A. et al. [19], conducted using the Brock classification criteria, observed that 42% of the patients examined had Brock grade ≥1 hearing loss. Of the patients, 28% experienced moderate to severe Brock grade ≥2 ototoxicity, and 12% of the patients studied required hearing aids after completion of cisplatin treatment. We also tried to observe the impact that non-genetic factors could have on the patients studied. The study was done on 102 children and adolescents who finished treatment with cisplatin. The average age of the children who had hearing loss was 7.8 years, which reveals that the lesions are even more important the younger the age. Males led the ranking, with injuries being more common in boys than in girls. Male patients with hepatoblastoma treated with cisplatin had the highest ototoxicity compared to male patients with other cancers, and an influence was also observed depending on the type of cancer. In addition, the mean cumulative dose of cisplatin was higher for patients who had ototoxicity, with a mean of 429 ± 78 mg/m^2^ [19].

#### 3.2.2. Concomitant Administration of Other Ototoxic Drugs

There are several drugs with an ototoxic effect that, when administered together with cisplatin, increase the risk of developing ototoxicity in treated patients. These patients can develop forms of severe ototoxicity, so it is important to know the potential associations that are harmful to hearing [14,16,61]. Concomitant administration of other ototoxic drugs significantly increases the risk and severity of cisplatin-induced ototoxicity, as they may act synergistically or additively on the cochlea. Ototoxic drugs used together with cisplatin can amplify oxidative stress and increase the accumulation of cisplatin in the cochlea. Together, they cause severe damage to the outer hair cells and stria vascularis. Synergistic administration of several ototoxic drugs activates inflammatory and apoptotic pathways, such as those mediated by caspase-3 and Bcl-2, helping to accelerate the apoptosis process [62,63].

Among the drugs known to have ototoxic potential are aminoglycosides, loop diuretics, vancomycin, quinine, and other compounds from the platinum derivatives family. Aminoglycosides affect the outer hair cells by accumulating in the endolymphatic fluid, amplifying oxidative stress at this level. This happens especially when they are administered for a longer period. There are studies that support that aminoglycosides can have a synergistic effect with cisplatin, leading to hearing loss, especially on high frequencies [64]. Loop diuretics affect ion transport in the vascular stria, which is responsible for maintaining homeostasis in the inner ear. In combination with cisplatin, this process can be amplified, leading to imbalances that damage cells at this level [65,66]. Quinine affects cellular function by altering the electrical potential of cochlear cell membranes. In combination with cisplatin, it can worsen hearing loss and vestibular dysfunction [67].

Unlike cisplatin, carboplatin is supposed to have much milder ototoxic effects. There are studies that have shown that pediatric patients treated with carboplatin alone at standard doses did not experience hearing loss or had minimal impairment. Therefore, carboplatin could be used as a treatment alternative to cisplatin, once the ototoxicity of the latter has been confirmed [17,62].

There are studies that do not find a link between the association of vancomycin, amphotericin B, or furosemide with cisplatin that amplifies the ototoxic effect of the latter. However, the combination of cisplatin with carboplatin contributed to a higher risk of hearing loss compared to the risk of patients treated with cisplatin alone [19,68]. Close monitoring and prevention of cumulative exposure are the most effective strategies to limit these effects.

#### 3.2.3. Cisplatin Doses

The dose of cisplatin administered directly influences the rate of occurrence, severity, and progression of ototoxicity due to the cumulative effects and the high concentration of the drug in the endolymphatic fluid at the cochlear level. Cisplatin generates oxidative stress, DNA damage, inflammation, and apoptosis, and these processes are more intense and frequent at higher doses or when the cumulative dose exceeds certain thresholds. The risk of ototoxicity in cisplatin treatment increases with the dose administered per session (single dose) and with the cumulative dose (total cisplatin administered during treatment). The administration of high doses in a single session causes an increased concentration in the endolymphatic fluid, which amplifies the oxidative processes in the cochlea. This effect is often seen in intensive treatment regimens (e.g., high-dose chemotherapy for advanced cancers). High cumulative doses lead to irreversible damage to external hair cells, stria vascularis, and auditory neurons. Cumulative effects become more apparent in patients treated for long periods or with multiple chemotherapy plans [1,19,62].

High doses of cisplatin or cumulative exposure above the threshold causes excess ROS production, thus exceeding the natural detoxification capacity of cochlear cells. This will lead to direct damage to cellular DNA, blocking replication and transcription, irreversible processes for many cells at the cochlear level. High doses will also stimulate the excessive production of proinflammatory cytokines, leading to mitochondrial damage and apoptosis. Cisplatin disrupts the function of the stria vascularis, which leads to the loss of ionic homeostasis necessary for the functioning of auditory cells [69].

There are studies that have highlighted the link between cisplatin-induced ototoxicity and cumulative doses of this drug [52]. The risk of developing moderate or severe ototoxicity was even higher as the cumulative dose of cisplatin exceeded 400 mg/m^2^. At this dose, hearing loss is observed, especially at high frequencies (>4 kHz). In addition, the risk of ototoxicity increased by 5–7% for each 100 mg/m^2^ dose of cisplatin. Regimens involving lower single doses (e.g., 20–40 mg/m^2^) have a lower risk, but ototoxicity can still occur with cumulative exposure. Cumulative doses above 600 mg/m^2^ are associated with a very high risk of severe and irreversible hearing loss [1,14]. Bertolini et al. [62] found a Brock grade hearing loss level ≥2 in 11% of patients treated with cisplatin, which progressed within 2 years of the end of therapy. There is other evidence to support that patients acquire hearing loss over time after completing treatment with cisplatin or that the degree of hearing loss increases with the passage of time [62]. Therefore, even minimal or mild hearing loss during cisplatin treatment can have complications with aging and affect quality of life.

#### 3.2.4. Head and Neck Radiotherapy

**Figure 3:**
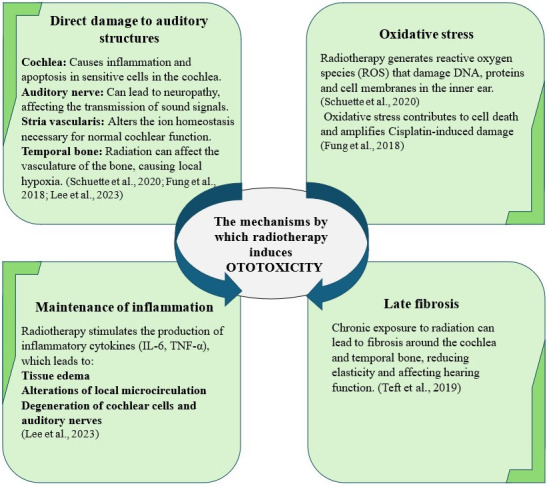
The main mechanisms by which radiotherapy induces ototoxicity [70,71,72,73].

Head and neck radiotherapy can significantly contribute to the development of ototoxicity, especially when used in combination with cisplatin. Both treatments contribute to the deterioration of the auditory structures, enhancing each other. Radiotherapy affects tissues by generating oxidative stress and maintaining inflammation, which amplifies the effects of cisplatin on the cochlea and associated structures [70]. Radiotherapy can reduce the ability of cells to regenerate and their vascularization, which makes the damage caused by cisplatin more difficult to repair. Head and neck radiotherapy in children under 5 years old can influence the onset of ototoxicity, especially since young age is a risk factor that should not be neglected [14]. Figure 3 illustrates the main mechanisms by which radiotherapy induces ototoxicity.

The maximum dose of head and neck radiotherapy depends on several factors such as the location of the tumor, its size, the patient’s age, and the critical structures that need to be protected. At the cochlear level, the tolerated dose is generally limited to 40–45 Gy to prevent the occurrence of ototoxicity. At the level of the auditory nerve, doses above 50 Gy can increase the risk of auditory neuropathy. [70,71] At the level of the temporal bone, doses above 60 Gy can cause fibrosis and tissue hypoxia, increasing the risk of hearing loss. The total dose administered in multiple fractions (e.g., 1.8–2 Gy per day) is usually limited to 70 Gy for radical treatments for advanced cancers. When radiotherapy is combined with cisplatin, the risk of ototoxicity is greatly amplified due to the synergistic effects [70,72,73].

#### 3.2.5. Renal Function

Renal function plays a very important role in cisplatin treatment because this drug is metabolized and eliminated mostly through the kidneys. When the kidneys function normally, they can effectively eliminate cisplatin and its metabolic products from the body. However, when renal dysfunction is present, the renal clearance of cisplatin may be compromised, leading to increased accumulation of the drug and an increased risk of toxicity [74]. When cisplatin levels are elevated in the blood and body fluids, the risk of ototoxicity is increased. Cisplatin can affect hearing cells and nerve cells in the inner ear in a similar way to kidney cells, causing dysfunction. Thus, compromised renal function may contribute to the onset or exacerbation of cisplatin-induced ototoxicity [19,69,75].

When cisplatin levels are increased in the body, the risk of ototoxicity may be amplified. Cisplatin can directly affect the auditory hair cells in the inner ear and the auditory nerve, causing structural and functional damage. Renal dysfunction can intensify these effects by increasing the concentration of cisplatin in the inner ear and hearing tissues, which can lead to further deterioration of hearing function. It is also believed that kidney dysfunction can worsen ototoxicity through inflammation and oxidative stress [74]. Hydration increases diuresis, and this is thought to reduce cisplatin-induced nephrotoxicity. It is important to assess patients’ renal function before starting treatment with cisplatin and to monitor this regularly during treatment. Patients with pre-existing renal dysfunction may require dose adjustments or longer intervals between doses to minimize the risk of renal toxicity [69].

#### 3.2.6. Nutritional Status

Poor nutritional status, characterized by vitamin, mineral, or antioxidant deficiencies, can amplify oxidative stress, inflammation, and cell damage caused by cisplatin. On the other hand, certain foods or nutrient compounds may provide protection against ototoxicity by reducing oxidative stress and supporting cell regeneration. Protein malnutrition can affect the synthesis of enzymes with antioxidant potential, which accelerates the formation of ROS and the degradation of cellular DNA [76,77]. The deficiency of antioxidants (vitamins A, C, and E, zinc, and selenium) reduces the body’s ability to neutralize ROS and intervene to stop inflammation, which amplifies apoptosis at the cochlear level [78]. Regarding obesity, the chronic inflammation associated with it can sensitize cochlear cells to the oxidative and inflammatory damage caused by cisplatin [79]. Inadequate hydration can affect ionic homeostasis and blood circulation in the cochlea, amplifying cisplatin accumulation and ototoxicity [80]. Table 2 lists the foods that can amplify the ototoxicity process and the foods that can prevent it.

In patients treated with cisplatin, it is advisable to avoid foods that could amplify the inflammatory process and to initiate a Mediterranean diet, based on antioxidants, fiber, and omega-3 fatty acids, along with a diet rich in fresh fruits and vegetables, whole grains, lean proteins, and essential fats [78,81]. In some situations, supplements of vitamins C and E, zinc, and selenium can be chosen and used as adjuvants to prevent ototoxicity under the supervision of a doctor [76,80]. Personalized dietary interventions, tailored to the patient’s needs, can reduce the risks and improve quality of life during cisplatin treatment.

## 4. Limitations of Genetic and Non-Genetic Factors: Discussion

The aim of our article was to highlight the main genetic and non-genetic factors discussed over time and to open new perspectives for future studies that could clarify research directions. In the future, we aim to conduct a systematic review to closely analyze all the uncertainties between studies and identify specific explanations as well as potential solutions. The role of genes in the process of ototoxicity is extremely important; however, it is crucial to identify the key genes on which genetic screening should focus, a task that is complicated by contradictory studies and differing opinions. Why does this happen? Some studies find an association between a specific gene and the ototoxicity process, while others fail to establish this correlation or associate the gene with only a minimal risk.

One key factor to consider is sample size. Studies with smaller sample sizes may lack the statistical power needed to detect subtle associations. A larger number of participants or a more specific population (e.g., patients with a certain type of cancer or a particular age group) can help identify clearer effects.

Additionally, the applied methodology must be carefully observed. The measurement of ototoxicity can be influenced by variability in assessment methods. As mentioned, there are multiple scales and classification criteria for interpreting ototoxicity (the Brock classification, Muenster classification, Boston classification, Chang classification, ASHA classification, CTCAE classification, and Standard National Cancer Institute classification), which may contribute to differing results. Other reasons for inconsistent findings include the impact of non-genetic factors, which introduce specific variables that can influence the results, as well as inter-individual variability. In studies on cisplatin-induced ototoxicity, various scales for classifying hearing loss can significantly influence the results. Each system has its advantages and disadvantages, and the use of one or another can lead to discrepancies in study outcomes.

**The Brock classification** focuses on hearing loss at high frequencies (4000–8000 Hz), which is relevant for cisplatin-induced ototoxicity. It is particularly applicable to children and is one of the most widely used scales in pediatric oncology. Its advantages include high sensitivity to cisplatin-induced ototoxicity and early detection of hearing impairment, as it is standardized and used in numerous clinical studies. Among its disadvantages are its limited applicability to adults, the potential overestimation of ototoxicity due to its reliance on high frequencies, which can also be affected by other variables, and the fact that it does not account for hearing loss at conversational frequencies (500–3000 Hz), which are essential for communication [82].

**The Chang classification** was developed as an alternative to Brock for assessing hearing loss in children, considering both high frequencies and conversational frequencies. It uses four severity grades based on the hearing threshold at 500–4000 Hz. It is more closely correlated with the clinical impact of hearing loss and provides a more balanced assessment than Brock, as it includes the frequencies essential for speech. However, it may underestimate early hearing loss as it places less emphasis on frequencies above 4000 Hz. It is not widely used in all studies, making comparisons more challenging [83].

**The Boston classification** focuses on hearing loss at high frequencies but also considers the impact on speech. It is primarily used in children to predict communication difficulties. It includes both high-frequency hearing loss and hearing loss that affects communication. This makes it potentially more relevant for assessing the clinical impact on patients. However, it is less widely used than Brock or Chang, making it more difficult to compare across studies, and it is less sensitive to early hearing loss [84].

**The Muenster classification** was developed to assess ototoxicity in children, considering both ears and the progression of hearing impairment. It classifies hearing loss into four grades, with an emphasis on frequencies between 1000 and 8000 Hz. It is more accurate than the Brock classification for evaluating overall auditory function and allows for the analysis of progressive hearing loss. However, it is less widely used, making comparisons with other studies more difficult, and it may not be sensitive enough to detect subtle high-frequency hearing losses [85].

**The ASHA classification** (American Speech-Language-Hearing Association) defines ototoxicity based on changes in hearing thresholds, rather than just as an absolute value. It considers a loss of ≥20 dB at one frequency, ≥10 dB at two consecutive frequencies, or hearing loss at three new frequencies as ototoxicity. It is more sensitive than other scales in detecting small changes that may indicate the onset of ototoxicity. It can be used for both children and adults. However, its disadvantages include the possibility that the established thresholds may be too sensitive, leading to an overestimation of ototoxicity, and it is more difficult to apply in large studies because it requires a comparison with the baseline audiogram [86].

**The CTCAE classification** (Common Terminology Criteria for Adverse Events) is used in oncological clinical studies to classify the adverse effects of treatments. Ototoxicity is classified into five grades based on its impact on communication and the need for interventions. Advantages include its standardization in oncology, which allows for comparisons between clinical studies, and its inclusion of the functional impact on the patient. However, it may underestimate early hearing impairment and is not specific to cisplatin- or drug-induced ototoxicity [87].

The differences between classification systems explain why studies on cisplatin ototoxicity can yield contradictory results. The choice of a scale can either underestimate or overestimate the prevalence of ototoxicity, influencing conclusions about the genes involved, safe cisplatin doses, and the effectiveness of preventive measures. Sensitivity and specificity vary because some scales (e.g., ASHA) detect even minor changes, while others (e.g., CTCAE) classify ototoxicity only at more severe levels. Scales such as Brock and Muenster are developed for children, while CTCAE and NCI are used in adult clinical studies, leading to differences in the populations studied. Some classifications (e.g., Brock) focus on high frequencies, while others (e.g., Chang) include conversational frequencies, which can alter the reported prevalence of ototoxicity. Some scales consider only an absolute change in hearing threshold, while others consider relative changes compared to the baseline audiogram, leading to different interpretations of hearing loss.

The correct choice of a classification scale is essential for the comparability of results between studies and for the validity of conclusions regarding Cisplatin ototoxicity. Some scales are more sensitive and relevant for certain populations, while others are less precise or too general. To decide which scales should be used more frequently and which should be avoided, the following factors must be considered: sensitivity to early hearing loss (the importance of high frequencies), clinical relevance (impact on communication and the need for interventions), standardized use in studies (ease of comparing results), and adaptability to children vs. adults. Based on the advantages and disadvantages, we believe that the most relevant classifications would be Brock, Chang, and ASHA. Of course, further studies are needed to determine which classification is optimal and when it should be used.

Some studies suggest a significant association with a specific gene, meaning that the effect is more evident in certain patient subgroups or may depend on other factors. For example, greater genetic susceptibility may be observed in patients of a particular age, sex, or those undergoing additional treatments. Each study may include a different population with distinct genetic, ethnic, and clinical factors, which can influence the results. For instance, an Asian population may have a different genetic profile compared to a European population, and these differences can impact susceptibility to ototoxicity. While some researchers use detailed audiometry to assess hearing loss, others may rely on simpler evaluations, which can lead to less precise identification of ototoxic effects.

Genetic interactions can also contribute to contradictory results. Cisplatin-induced ototoxicity is a complex phenomenon in which multiple genes and environmental factors play a role. Thus, the effect of a genetic variant may be more evident in the presence of other polymorphisms or specific clinical factors. For a clearer understanding, additional studies with a robust design and simultaneous analysis of multiple genes involved in cisplatin metabolism would be necessary.

It is true that genetic studies investigating the association between genotypes and the risk of ototoxicity can lead to contradictory or even false-positive results. Additionally, in a field with numerous implicated genes, questions arise regarding which genes should be prioritized in research. Studies with larger sample sizes and adequate statistical power are much more reliable. If a study finds a significant association in a small group of participants, it may be a coincidence or a statistical error. In contrast, multicenter studies or those replicated in another participant cohort are more credible. A valid and credible study is one that can be replicated by other researchers in different contexts or populations. Replication studies are essential for establishing the validity of a genetic association. It is important to assess whether the method used to identify genetic associations is robust and correctly applied. Studies that utilize advanced techniques for correcting multiple comparison errors (e.g., the false discovery rate—FDR) and control for confounding variables are generally more reliable.

Another important factor is the generalizability of results. If a genetic polymorphism is associated with a particular phenomenon in a specific population (e.g., a group of cancer patients), it must be tested in other groups to determine if it is also valid in different populations. Studies published in high-impact journals (recognized journals in the field) and subjected to a peer-review process have a higher chance of being credible. When many variables (genes and polymorphisms) are tested simultaneously—as is the case in large-scale association studies or genome-wide association studies (GWAS)—there is a higher probability of finding associations that are coincidental. This phenomenon is known as a “Type I error” (false positive). Studies with positive or significant results are more frequently published than those with negative or neutral results. This phenomenon, known as “publication bias”, can lead to an overestimation of the relevance of a genetic association.

Genes involved in key biological processes related to drug metabolism and transport are the most relevant. For example, genes involved in cisplatin metabolism, such as ***GSTT1***, ***GSTM1***, ***SLC22A2***, and ***ABCC3***, or those involved in the production of reactive oxygen species (***SOD2*** and ***CAT***), are usually prioritized because they can directly influence the accumulation of cisplatin in the cochlea. Genes that have been consistently associated with ototoxicity across multiple studies, regardless of population, will be prioritized for further investigation. Additionally, genes that have been replicated in various studies are more credible and should be prioritized. Genes involved in a patient’s response to treatment and those that directly impact the risk of ototoxicity will also be prioritized. These genes could help develop genetic tests to identify patients who are more susceptible to cisplatin’s adverse effects and allow for treatment adjustments accordingly. To prioritize genes in the context of ototoxicity, it is crucial to consider functional evidence, result replicability, and the clinical impact of the association. Genes with a well-defined biological mechanism and consistent findings across multiple studies are the most promising candidates for further research.

A high-priority gene in the context of ototoxicity is ***ACYP2 (rs1872328)***. This is one of the genes most strongly associated with cisplatin-induced hearing loss. The studies we have referenced consistently find a clear association with ototoxicity, and the strength of the existing evidence is a key reason for its prioritization. For example, ***ACYP2 rs1872328*** has been consistently linked to ototoxicity across multiple studies, whereas ***SLC16A5 rs4788863*** has shown less consistent results, being significant only in certain cohorts [27]. Genes directly involved in cisplatin metabolism, detoxification, drug transport, or auditory cell protection are considered more relevant. For instance, ***GSTT1*** and ***GSTM1*** play a role in cisplatin detoxification and have been linked to ototoxicity in multiple studies. In contrast, ***COMT rs9332377***, which is involved in dopamine metabolism and oxidative stress regulation, has a less certain mechanism regarding its role in ototoxicity, with studies being contradictory.

The impact of genetic effects is another key factor in prioritizing genes. Genes with a strong influence on ototoxicity risk are given higher priority than those with minor effects. Measures such as the odds ratio (OR) and hazard ratio (HR) are used to assess the impact of each polymorphism. For example, ***ACYP2 rs1872328*** has a high OR (>2 in some studies), indicating a significant impact on ototoxicity risk [36,37], whereas ***GSTP1 rs1695*** has weaker effects, with some studies finding no association at all [15,28].

If a gene is associated with other cisplatin-induced toxicities (e.g., nephrotoxicity and myelosuppression), it suggests a central biological role. ***ABCC3 rs1051640***, for instance, is involved in both ototoxicity and cisplatin elimination from cells, making it an important genetic marker [15]. Some genes could be used for genetic screening or personalized treatment. Markers that can influence clinical decisions are more valuable. ***TPMT rs12201199***, ***rs1142345***, **and *rs1800460*** are already used in other clinical contexts (e.g., thiopurine metabolism), increasing their potential application in oncology [48]. The *SOD2 rs4880* also shows an association with ototoxicity in the studies mentioned by us, but it does not have immediate applicability, as further studies are needed in this regard [15,27,38].

The best-supported genetic markers based on current evidence are ***ACYP2 rs1872328***, which has a strong association and a significant effect; ***GSTT1/GSTM1***, which has a well-documented role in cisplatin detoxification; ***TPMT rs12201199***, ***rs1142345*, and *rs1800460***, which are already used clinically in other fields; and ***ABCC3 rs1051640***, which is involved in cisplatin transport and is a potential therapeutic biomarker.

Bias in genetic studies on ototoxicity can influence the results and lead to false-positive or false-negative associations. It can arise from various sources, affecting the credibility and reproducibility of findings. **Selection bias** can contribute to numerous errors, leading to contradictory results, and some genes may appear more important than they are. For example, studies on ***ACYP2 rs1872328*** have been mainly conducted on children, and the results may not apply to adults.

Let us examine a few articles from those we selected regarding certain genes. For ***GSTM1 del***, the studies mentioned [15,24,27,28,30] found more or less significant associations with cisplatin-induced ototoxicity. For ***GSTT1 del***, some studies found more or less relevant associations with ototoxicity [15,28,30], while others found a significant association [27,31], and one study found an association with otoprotection [24]. For ***GSTP1 rs1695***, we have studies that support the ototoxic effect of this polymorphism [14,27], and studies that associate it partially or not at all with ototoxicity [15,28].

Lui et al. (2018) [27] conducted a study investigating several genetic polymorphisms in a pediatric population of 106 children treated with platinum derivatives, excluding cranial irradiation, in a retrospective design. The Brock criteria were used for the analysis. Sixty patients received cisplatin, 10 received carboplatin, and 36 received both cisplatin and carboplatin. The median cumulative dose of cisplatin was 400 mg/m^2^. The children were treated for osteosarcomas (11%), retinoblastomas (8%), hepatoblastomas (18%), neuroblastomas (27%), or malignant germ cell tumors (35%).

Barahmani et al. (2009) [30] investigated the relationship between *GSTM1* and *GSTT1* polymorphisms, survival, and toxicity in 42 children with medulloblastoma diagnosed and treated at Texas Children’s Cancer Center. Kaplan–Meier analysis was conducted to determine if *GST* polymorphisms were associated with progression-free survival (PFS), and logistic regression was performed to explore associations between *GST* polymorphisms and the occurrence of grade 3 or higher myelosuppression (≥ grade 3), ototoxicity, nephrotoxicity, neurotoxicity, and intellectual deficiency. All patients older than 3 years at diagnosis were treated with craniospinal radiation followed by systemic chemotherapy. Patients younger than 3 years were initially treated with systemic chemotherapy, followed by craniospinal radiation when they reached 3 years of age or relapsed. Most patients received four cycles of high-dose chemotherapy. A multiplex polymerase chain reaction (PCR) technique was used to amplify both *GSTM1* and *GSTT1* simultaneously in a single PCR reaction.

In the two studies presented as examples, the selection criteria are different, which may lead to contradictory results, as indeed happened. In the study by Barahmani et al. (2009) [30], there may also be a confounding bias. A medulloblastoma is treated with cranial radiation therapy, which can induce ototoxicity independent of cisplatin. If this aspect was not controlled, it could alter the results. There could also be a publication bias as the study includes several polymorphisms but reports only the significant results, which may overestimate the effect of the genes analyzed. The results are relevant but need to be confirmed in studies that more strictly control for confounders (e.g., radiation therapy).

In the study by Lui et al. (2018) [27], there may be a confounding bias if environmental factors (e.g., noise exposure and administration of protective agents) were not controlled for, as they may influence the results. Additionally, it is possible that only genes with significant associations were reported, creating a risk of overreporting positive results. The study provides valuable information, but replication in larger cohorts with stricter control of confounding variables is needed. A bias related to sample size may also appear. One study was conducted on 106 children, and the other on 42. If the number of participants is too small, the results may be unstable and cannot be generalized, and the reduced statistical power increases the risk of false positives and false negatives.

If we were to conduct a thorough analysis of all the mentioned articles, we would likely discover many discrepancies and identify which articles are the most relevant and could provide a solid foundation for future studies.

Can we conclude that a gene is a real risk factor? Not if the evidence is weak or contradictory. A single significant association in a small study is not enough to confirm that a gene is a real risk factor. However, we can if there are consistent replications in multiple independent studies, if meta-analyses are conducted that combine data from different studies to increase the statistical power, and if functional studies are carried out that demonstrate clear biological mechanisms through which the gene influences cisplatin toxicity. For a gene to be considered a real risk factor, more than just a statistical association is needed. The evidence must meet the following criteria:

**Replicability**: Associations must be demonstrated in multiple independent studies with robust methodologies. If a polymorphism is associated with ototoxicity in only one study or in a very small sample, the chances of it being a coincidence are high.

**Consistency across populations**: If an association is only valid in one population (e.g., Asians but not Europeans), it may indicate either a real effect dependent on genetic background or a methodological issue (e.g., population stratification bias).

**Functional studies**: There must be a clear biological mechanism explaining why the polymorphism affects ototoxicity. ***ACYP2 rs1872328*** → Functional studies suggest that it affects the apoptosis of ciliated cells in the inner ear. ***SLC22A2 rs316019*** → This alters the transport of cisplatin into cells, affecting its accumulation in the inner ear. ***GSTM1/GSTT1*** → This is involved in oxidative metabolism and the detoxification of cisplatin.

**Meta-analyses**: Individual studies may have contradictory results. A meta-analysis combining data from multiple studies can more clearly evaluate the effect of a gene on the risk of ototoxicity. If meta-analyses do not support a clear association, the evidence is weak.

**Predictive power**: Can this polymorphism be used to predict ototoxicity in a large population? If the risk difference between genotypes is small, the clinical impact is insignificant.

***ACYP2 rs1872328*** has been associated with cisplatin-induced ototoxicity in multiple studies. Meta-analyses and functional studies suggest a plausible biological mechanism. This gene warrants further research. On the other hand, polymorphisms such as ***GSTM3 rs1799735*** have mixed results and are not supported by strong functional studies, making them less of a priority. Therefore, we encourage future studies to focus on identifying real genetic factors that can be integrated into national screening programs to reduce hearing loss and ototoxicity.

Although genetics plays a role in individual susceptibility to the toxic effects of cisplatin, there are several limitations that make it difficult to use genetic polymorphisms as reliable biomarkers. Ototoxicity is influenced not only by genetic variations but also by factors such as cisplatin dosage, treatment duration, noise exposure, and other environmental factors. Typically, each genetic variant contributes only a small percentage to the overall risk. Ototoxicity is a complex phenomenon in which multiple genes interact with non-genetic factors. There is no single polymorphism that fully explains ototoxicity. For example, ***ACYP2 rs1872328***, ***SLC22A2 rs316019***, ***GSTP1 rs1695***, and ***TPMT rs12201199*** may be associated. Although some polymorphisms are linked to ototoxicity, there is still no clinically recognized genetic test available for prevention. Even combining the most powerful identified genetic factors cannot accurately predict ototoxicity in an individual. Even if we know that someone has an increased genetic risk, there are no proven therapies to prevent ototoxicity based on their genetic profile [1,2,14].

Non-genetic factors play a crucial role in cisplatin-induced ototoxicity and can explain why there are significant variations between patients, even in the presence of genetic polymorphisms associated with increased risk. In many cases, these factors have a greater impact than genetics and can influence the onset and severity of ototoxicity. Some patients who receive high doses of cisplatin do not develop ototoxicity, while other patients who receive low doses may develop it. This phenomenon can be explained by the complex interaction between genetic and non-genetic factors that determine an individual’s susceptibility to cisplatin-induced ototoxicity. There is no single determinant factor, but rather a combination of mechanisms that influence each patient’s response to treatment. Ototoxicity depends not only on the dose but also on the complex interaction between genetics, metabolism, clinical factors, and environmental factors. Focusing solely on genetics may be a misguided approach if non-genetic factors are not also considered. Personalizing treatment based on a combined profile (genetic, clinical, and environmental) is essential for more accurate predictions of the risk of ototoxicity [2,14,15].

Although non-genetic factors play a crucial role in determining the risk of cisplatin-induced ototoxicity, they also have significant limitations that can affect the accurate assessment and management of the patient. Significant inter-individual variability must be considered. For example, two patients receiving the same dose of cisplatin under the same conditions may respond differently based on their individual characteristics. This could be due to differences in each person’s physiology or immune responses, making accurate predictions based on non-genetic factors difficult [14,62]. Patients who receive high doses and do not develop ototoxicity may have a faster elimination process for cisplatin (their kidneys efficiently excrete the drug, reducing the exposure time of the inner ear to toxicity), efficient antioxidant systems that neutralize the oxidative stress caused by cisplatin, or efflux transporters (e.g., SLC22A2 and ABCC3) that help eliminate cisplatin from the cells of the inner ear. Each factor (e.g., comorbidities, associated medications, or noise exposure) can interact with others in an unpredictable way, and these interactions can vary greatly from one patient to another [42,49,74].

Measuring and controlling non-genetic factors is very difficult. In some cases, patients may receive variable doses of cisplatin, and the administered dose is not always well-monitored or -standardized. Additionally, different administration protocols can affect the exposure time and distribution of the drug in the body [62]. Exposure to noise or other toxic agents is not always quantifiable with precision and can vary during the treatment. Moreover, living environments (e.g., home or workplace) may influence the risk of ototoxicity, but are difficult to control or standardize in a clinical setting. The impact of comorbidities on treatment management should not be overlooked either [2,16]. Comorbidities (e.g., diabetes, hypertension, and renal insufficiency) can influence the risk of ototoxicity, but each condition adds a layer of complexity. For example, patients with renal insufficiency may eliminate cisplatin more slowly, potentially exacerbating toxic effects, but treatments for comorbidities can interfere with the efficacy and safety of cisplatin. The administration of drugs that may increase the risk of ototoxicity (e.g., aminoglycosides and loop diuretics) or that may alter cisplatin elimination adds an additional layer of uncertainty and complexity in managing side effects [14,16,74]. Non-genetic factors cannot be viewed in isolation, as they can interact with genetic variables in ways that are difficult to predict or control. For example, a person with a polymorphism that reduces the ability to detoxify free radicals (such as GSTP1 or SOD2) may be more sensitive to even low doses of cisplatin, even in the absence of significant non-genetic factors (e.g., comorbidities or noise exposure).

Non-genetic factors have a significant impact on cisplatin-induced ototoxicity but measuring and controlling them remains a major challenge. While they are essential for understanding individual risk, these factors are difficult to standardize and can vary significantly between patients. Moreover, their interactions with genetic factors can further complicate the identification of a clear and predictable risk of ototoxicity. An integrated approach, combining both genetic and non-genetic factors, is essential for a more accurate and personalized assessment of ototoxicity risk.

The credibility of the idea that genes are risk factors for cisplatin-induced ototoxicity is supported by multiple layers of evidence but also faces several important limitations. There is unexplained inter-individual variability. As previously mentioned, some patients develop severe ototoxicity at low doses of cisplatin, while others experience no adverse effects even at high doses. This variability cannot be fully explained by non-genetic factors (such as age, dosage, comorbidities, etc.), suggesting a potential genetic influence.

Another possible explanation could be related to the role of genes involved in cisplatin metabolism. Many genes associated with detoxification (e.g., *GSTM1*, *GSTT1*, and *GSTP1*), transport (e.g., *SLC22A2* and *LRP2*), or DNA repair (e.g., *XPC* and *XPD*) directly affect how the body processes cisplatin. Polymorphisms in these genes may lead to functional variations (such as slower enzymatic activity, reduced transport capacity, or diminished DNA repair efficiency), which may theoretically increase the risk of toxicity.

If a single-nucleotide polymorphism (SNP) is clearly associated with an increased risk, it could potentially be used for risk stratification or to guide the selection of alternative therapies. However, caution is warranted, as association does not imply causation. The statistical association of an SNP with ototoxicity does not necessarily mean it causes the condition—it may simply be a genetic marker linked to another causative variant. Many studies have reported conflicting results (for example, *GSTP1 rs1695* is clearly associated in some studies and not at all in others), likely due to small sample sizes, differences in ethnic backgrounds, the use of diverse classification scales, and other variables. Moreover, issues such as a publication bias toward positive findings, the lack of adjustments for multiple comparisons, and non-random patient selection can all contribute to false-positive results.

Genetics offers a promising avenue of investigation, but we currently lack robust and consistent evidence that any specific gene is a definitive causal factor. At this stage, these genetic variations should be viewed as susceptibility factors, not absolute predictors. Genetic studies provide moderate evidence, with some identifying significant associations between genetic variants and ototoxicity, while others do not. Cisplatin induces oxidative stress, DNA damage, and inflammation, processes in which genes such as *GSTs*, *SOD2*, *XPD*, *ACYP2*, and *TPMT*, among others, are involved. There is a plausible molecular biology rationale supporting the idea that genetic polymorphisms may influence how effectively a cell responds to these stressors. These genes have the potential to act as risk factors, but they cannot yet be used independently to predict ototoxicity. Genetics should neither be disregarded nor overemphasized. Genes should be viewed as modulators of risk, rather than absolute determinants. There is an urgent need for large, multicenter, standardized studies to replicate and validate existing findings. Until such data are available, genetics remains a complementary, not a definitive, tool.

Studies supporting a gene-centered view of cisplatin-induced ototoxicity are important and promising, but they face several methodological, conceptual, and clinical challenges that limit the validity and applicability of their conclusions. Among the most relevant issues identified in these studies are the small sample sizes (most include fewer than 200 patients, and some even fewer than 100), leading to a higher likelihood of false-positive or false-negative results. Consequently, observed associations may be incidental and not reproducible in other cohorts.

Another significant concern is the lack of reproducibility. Studies conducted in different populations and across various ethnic backgrounds, age groups, or cancer types often yield conflicting results. Without cross-population validation, the generalization of findings remains limited. Additionally, the tendency to focus on individual genes represents a reductive and potentially biased approach that overlooks the complexity of genetic interactions. A more comprehensive and hypothesis-free alternative would be genome-wide association studies (GWAS), which are better suited to capturing the polygenic nature of susceptibility. Methodological biases also need to be systematically addressed across studies to ensure the consistency and reliability of findings.

Lastly, an exclusive focus on genetic factors tends to underestimate the critical role of non-genetic variables (such as environmental exposures, comorbidities, or treatment regimens) that may significantly influence the risk of ototoxicity. Therefore, a balanced and integrative approach is essential in advancing our understanding of cisplatin-induced hearing loss.

If genes are not truly risk factors for cisplatin-induced ototoxicity (or if their role is significantly smaller than previously estimated), there are important implications for both research and clinical practice. If genes do not play a major role, genetic testing will not be able to effectively predict the risk of ototoxicity, and personalized preventive measures (e.g., dose reduction for “at-risk” patients) may be unjustified or even harmful, potentially compromising treatment efficacy. This could ultimately lead to wasted resources and ineffective interventions. Many genetic studies require substantial financial and logistical investment. If the research focus is placed on genes that are not genuinely involved, more relevant and promising avenues may be overlooked, resulting in fragmented and slow scientific progress.

Furthermore, if genes are not actual risk factors, genetic testing could become unnecessary. Patients might undergo costly tests with no practical benefit, potentially resulting in a false sense of security (if the test is negative) or unnecessary anxiety (if the test is positive but clinically insignificant). By focusing exclusively on genetic factors and neglecting other relevant risk variables, clinical practice risks becoming unbalanced and guided by an incomplete or incorrect model of causality. Additionally, if studies propose genotype-driven protective therapies (e.g., antioxidant treatment only for patients with specific variants), and these genes do not have a real impact, such interventions may fail, clinical trials may be invalidated, and trust in personalized medicine could diminish. Therefore, rigorous validation, replication of findings, and multifactorial research approaches are essential to ensure both scientific integrity and clinical utility.

Genetic testing in patients to identify the risk of cisplatin-induced ototoxicity can offer significant advantages, but it also comes with important limitations. Even though not all genes involved are completely clarified and there is no global consensus on definitive genetic markers, genetic testing can provide useful information for treatment personalization and risk management. Even without full agreement on all the genes involved, genetic testing can help identify patients at high risk of developing ototoxicity, allowing for dose adjustments of cisplatin or the choice of alternative therapies.

Testing can guide the selection of a less toxic treatment regimen, reducing the cisplatin dose for patients with genetic variants that predispose them to ototoxicity or avoiding its use entirely in cases with very high risk. Patients with a high genetic risk could benefit from more careful and frequent monitoring of hearing and renal function, enabling early detection of ototoxicity and preventing permanent damage. If genetic variants predisposing to ototoxicity are identified, doctors can intervene earlier by adjusting the treatment to prevent irreversible hearing loss. Knowledge of genetic risks can influence therapeutic decisions, providing physicians with a better framework for treatment planning. For example, certain drug combinations can be avoided, or additional measures can be taken to protect the inner ear. Genetic testing can also indicate whether certain ototoxic drugs (e.g., aminoglycosides) are riskier when combined with cisplatin for those patients, facilitating the selection of safer alternatives.

We must also highlight the negative aspects associated with genetic testing. There may be an incomplete association between genes and the ototoxicity process. Not all genes relevant to ototoxicity are known, and in many cases, associations are unclear or inconsistently replicated across different studies. Genetic testing might not be precise enough to predict adverse effects in all patients. Even with genetic testing, not all patients with the same genetic variants will develop ototoxicity. This can result in a low predictive value, reducing its overall usefulness.

Genetic testing can be expensive, and not all patients have access to such analyses, especially in vulnerable populations or regions with limited resources. In healthcare systems with tight budgets, genetic testing may become a complex and inaccessible task for patients, leading to inequalities in access to personalized treatments. Genetic testing may also cause anxiety or fear about potential risks. If patients learn they have a genetic predisposition to ototoxicity, this could affect their quality of life and influence their perception of future treatments. There are risks related to confidentiality and the potential misuse of genetic information, which could be used in a discriminatory manner (e.g., in health insurance or employment decisions). The analysis of genetic data is complex and requires specialized expertise to interpret the results accurately. Additionally, correlating genetic data with a patient’s clinical context (e.g., comorbidities and concurrent medications) can be challenging, complicating clinical decision-making.

Cisplatin is widely used in cancer treatment, but its associated toxicities, including ototoxicity, nephrotoxicity, and neurotoxicity, have driven researchers to explore alternative therapeutic options. Although various alternatives exist, each comes with its own advantages and challenges.

One such alternative is **carboplatin**, which has a similar mechanism of action to cisplatin but with reduced toxicity. It is commonly used in the treatment of ovarian and lung cancer. However, it is less effective than cisplatin in testicular cancer, where cisplatin remains the preferred treatment due to its superior efficacy [54,68]. Another option is **oxaliplatin**, which forms bonds with DNA and inhibits cell replication. Although it presents a lower risk of nephrotoxicity and ototoxicity compared to cisplatin, its main drawback is increased peripheral neurotoxicity, which can significantly affect patients’ quality of life. **nedaplatin** functions in a manner similar to cisplatin but with a milder toxicity profile, making it a potential alternative in specific cases [62,63].

Beyond platinum-based compounds, targeted therapies and immunotherapies are being explored as potential alternatives to cisplatin. Agents such as **bevacizumab**, which targets vascular endothelial growth factor (VEGF), and immune checkpoint inhibitors such as **pembrolizumab** and **nivolumab** (which block PD-1/PD-L1) have shown promising results in various cancers. Additionally, **erlotinib** and **gefitinib**, which are epidermal growth factor receptor (EGFR) inhibitors, provide targeted treatment options with encouraging outcomes. However, these therapies are often associated with high costs and limited accessibility, making them challenging to implement on a large scale [11,18].

Replacing cisplatin in clinical practice comes with significant challenges. One of the major concerns is the potential loss of efficacy, as some alternatives, such as carboplatin, may be less effective in treating certain cancers, including testicular, head, and neck cancers. Additionally, while alternative therapies may reduce some toxic effects, they can introduce new risks. For instance, oxaliplatin is associated with severe neurotoxicity, which can be debilitating for patients. Another concern is tumor resistance, as some cancers that respond well to cisplatin may not be as sensitive to alternatives such as carboplatin. Finally, newer targeted therapies and immunotherapies, while promising, are often significantly more expensive than conventional chemotherapy and may not be widely available to all patients [16,62].

In conclusion, despite its toxic effects, cisplatin remains one of the most effective chemotherapeutic agents for certain cancers. Finding an ideal replacement is challenging, and any alternative must be carefully chosen based on the type of cancer being treated, its toxicity profile, and the individual patient’s response to therapy.

Therefore, genetic testing has its place, and based on high-quality studies that identify real genes involved in the ototoxicity process, many complications can be avoided. Tested patients, especially children, can benefit from individualized treatments. Cisplatin doses can be adjusted, or it can be replaced with an alternative drug to prevent ototoxicity and its long-term consequences on hearing.

## 5. Conclusions

Genetic and non-genetic risk factors play an extremely important role in the occurrence of ototoxicity in pediatric patients treated with cisplatin. Determining risk groups could help individualize treatment protocols, prevent complications, and improve patients’ quality of life. Patients with certain genotypes could be followed closely throughout treatment to prevent or reduce adverse effects. Recent advances in the field of medical genetics make it possible and even encourage genetic screening in cancer patients to discover patients predisposed to chemotherapy ototoxicity. Identifying genetic polymorphisms that can determine vulnerability to chemotherapy-induced hearing loss could be an important step in establishing individualized therapeutic protocols so that effects on the inner ear can be prevented and limited.

The analysis of the data obtained in the different studies can be difficult because there is great variability in these data, which are explained using different ototoxicity grading scales, and due to the heterogeneity of the batches studied in terms of non-genetic factors. The use of a common rating scale in the various studies, as well as the organization of patient groups according to clear criteria, could provide much clearer and more clinically relevant conclusions.

If we refer to non-genetic factors, we must give importance to the age of the patient, because children and the elderly have some peculiarities that influence the action of cisplatin at the cochlear level. Although not many studies look at patients by gender, there is evidence that men are more susceptible to ototoxicity than women. The increase in total doses of cisplatin correlates directly with the severity of hearing loss, with damage to cochlear cells becoming irreversible at high doses. In this sense, adaptation of the minimum effective doses by the oncologist is of particular importance in avoiding excess ototoxicity. The concomitant administration of cisplatin with other medicinal products with ototoxic potential should be considered and avoided whenever possible. Antioxidant deficiencies or a diet low in protective nutrients can increase the risk of ototoxicity. Certain antioxidant-rich foods, such as fruits and vegetables, may provide protection against cisplatin-induced oxidative stress, while poor nutrition could amplify the damage.

Thus, effective management of the risk of ototoxicity in cisplatin treatment is possible, provided that a personalized approach considers both genetic and non-genetic factors. Identifying and minimizing these factors can help reduce hearing loss and improve the quality of life of patients treated with cisplatin.

## Figures and Tables

**Table 2:** Foods that can amplify the process of ototoxicity and foods that can prevent it.

Foods That Can Amplify the Process of Ototoxicity [76,78,79,80,81]	
FOOD	SOURCE
**Foods Rich in Salt** Excessive salt consumption can affect the ion balance in the inner ear, aggravating the damage caused by cisplatin.	Meats Processed food Snacks Cheese
**Saturated and trans fats** Diets high in saturated and trans fats may promote systemic inflammation and reduce antioxidant activity	**Saturated fats** Butter Fat cheeses Cream Fatty meat (pork, lamb) Bacon Meats Coconut/palm oil **Trans fats** Pastry products Fried products (fries, chips, fast food) Margarine Processed products
**Artificial additives** Some food additives, such as monosodium glutamate (MSG), can amplify oxidative stress in the inner ear.	Processed products Canned foods Packaged products
**Alcohol** Alcohol consumption may increase oxidative stress and inflammation, sensitizing the cochlea to the effects of cisplatin.	
**Foods that can prevent the process of ototoxicity [76,78,79,80,81]**	
**FOOD**	**SOURCE**
**Natural antioxidants** **Vitamin C** It neutralizes ROS and reduces oxidative stress in the cochlea. It increases the synthesis of glutathione, a powerful endogenous antioxidant. **Vitamin E** Protects cell membranes from lipid peroxidation. **Carotenoids** Beta-carotene and lycopene can reduce inflammation and oxidative stress.	**Vitamin C** Citrus fruits, tropical fruits, strawberries, kiwi, berries, sea buckthorn, melon, guava Bell pepper, tomatoes, zucchini, rocket, parsley, spinach, broccoli, cauliflower, cabbage **Vitamin E** Almonds, walnuts, peanuts Olive oil, sunflower oil, pumpkin seeds, sunflower seeds, grains Avocados, spinach, kale, parsley, mango, kiwi, salmon, trout, shrimp, Soy, peanut butter **Carotenoids** Carrots, spinach, tomatoes, pumpkin, sweet potato, red pepper, cabbage, lettuce, broccoli, parsley, apricot, papaya
**Minerals with an antioxidant role** **Selenium** This cofactor of glutathione peroxidase reduces ROS accumulation. **Zinc** Supports the functioning of antioxidant enzymes and cell regeneration.	**Selenium** Nuts, fish, seafood, Eggs, beef liver, turkey, pork, chicken Whole grains (brown rice, wheat, oats, rye) Yogurt Beans, lentils, chickpeas, mushrooms, garlic, onions **Zinc** Seafood Lamb, pork, beef, eggs (especially the yolk) Pumpkin seeds, sesame seeds, cashews, walnuts, almonds Lentils, beans, chickpeas, peas Whole grains Milk, yogurt
**Omega-3 fatty acids** They have anti-inflammatory properties that may reduce cisplatin-induced inflammation in the cochlea.	**Omega-3 fatty acids** Fatty fish, seafood, cod liver oil, fish oil Flax seeds, chia seeds, hemp seeds, walnuts, flax oil, hemp oil Soy, edamame, cabbage, spinach
**Polyphenols** **Resveratrol** Powerful antioxidant that can prevent cell death. **Curcumin** It reduces inflammation and protects against oxidative stress.	**Resveratrol** Grapes, blueberries, dark chocolate
**Sulfur-rich foods** They can increase the synthesis of glutathione and other antioxidant enzymes.	Garlic, onion

## Abbreviations

ACYP2	*Acylphosphatase 2 gene*
ABCC3	ATP-Binding Cassette Subfamily C member 3
ABCB5	ATP-Binding Cassette Subfamily B member 5
CTR1	Copper transporter 1
COMT	Catechol-O-methyltransferase
ERCC	Excision Repair Cross-Complementing Group
EPXH1	Epoxide hydrolase 1
GST	Glutathione S-transferase
GSTM1	Glutathion S-transferase Mu 1
GSTM3	Glutathion S-transferase Mu 3
GSTT1	Glutathion S-transferase theta 1
GSTP1	Glutathion S-transferase pi gene
LRP2	Low-density lipoprotein receptor gene
OCT	Organic cation transporter
P53	Tumor antigen p53
ROS	Reactive oxygen species
SLC16A5	Solute Carrier Family 16 Member 5
SLC22A2	Solute Carrier Family 22 Member 2
SOD	Superoxide dismutase
SNP	Single-nucleotide polymorphism
TPMT	Thiopurine S-methyltransferase
TNF-α	Tumor necrosis factor-α
XPC	Xeroderma pigmentosum complementation group C
XPD	Xeroderma pigmentosum complementation group D
